# *Bothrops jararaca* Venom Metalloproteinases Are Essential for Coagulopathy and Increase Plasma Tissue Factor Levels during Envenomation

**DOI:** 10.1371/journal.pntd.0002814

**Published:** 2014-05-15

**Authors:** Karine M. Yamashita, André F. Alves, Katia C. Barbaro, Marcelo L. Santoro

**Affiliations:** 1 Laboratory of Pathophysiology, Institute Butantan, São Paulo, São Paulo, Brazil; 2 Department of Clinical Medicine, School of Medicine, University of São Paulo, São Paulo, São Paulo, Brazil; 3 Immunopathology, Institute Butantan, São Paulo, São Paulo, Brazil; Universidad de Costa Rica, Costa Rica

## Abstract

**Background/Aims:**

Bleeding tendency, coagulopathy and platelet disorders are recurrent manifestations in snakebites occurring worldwide. We reasoned that by damaging tissues and/or activating cells at the site of the bite and systemically, snake venom toxins might release or decrypt tissue factor (TF), resulting in activation of blood coagulation and aggravation of the bleeding tendency. Thus, we addressed (a) whether TF and protein disulfide isomerase (PDI), an oxireductase involved in TF encryption/decryption, were altered in experimental snake envenomation; (b) the involvement and significance of snake venom metalloproteinases (SVMP) and serine proteinases (SVSP) to hemostatic disturbances.

**Methods/Principal Findings:**

Crude *Bothrops jararaca* venom (BjV) was preincubated with Na_2_-EDTA or AEBSF, which are inhibitors of SVMP and SVSP, respectively, and injected subcutaneously or intravenously into rats to analyze the contribution of local lesion to the development of hemostatic disturbances. Samples of blood, lung and skin were collected and analyzed at 3 and 6 h. Platelet counts were markedly diminished in rats, and neither Na_2_-EDTA nor AEBSF could effectively abrogate this fall. However, Na_2_-EDTA markedly reduced plasma fibrinogen consumption and hemorrhage at the site of BjV inoculation. Na_2_-EDTA also abolished the marked elevation in TF levels in plasma at 3 and 6 h, by both administration routes. Moreover, increased TF activity was also noticed in lung and skin tissue samples at 6 h. However, factor VII levels did not decrease over time. PDI expression in skin was normal at 3 h, and downregulated at 6 h in all groups treated with BjV.

**Conclusions:**

SVMP induce coagulopathy, hemorrhage and increased TF levels in plasma, but neither SVMP nor SVSP are directly involved in thrombocytopenia. High levels of TF in plasma and TF decryption occur during snake envenomation, like true disseminated intravascular coagulation syndrome, and might be implicated in engendering bleeding manifestations in severely-envenomed patients.

## Author Summary

Although the abundance of reports about hemostatic disturbances in snakebites, few studies have addressed how crude snake venoms evoke blood coagulation disturbances *in vivo*. Snake venoms contain several components that disturb hemostasis, and the prevailing model claims that coagulation disturbances observed in patients are triggered directly by those toxins. However, taking into account the physiological mechanisms that activate the coagulation cascade, tissue factor might also be generated and decrypted during snake envenomation. We investigated herein if tissue factor and protein disulfide isomerase, an enzyme that controls the encryption/decryption of tissue factor, were altered during experimental envenomation in rats. We observed increased activity/expression of tissue factor at the site of venom injection, as well as in lungs, and decreased expression of protein disulfide isomerase at the site of venom injection. Moreover, tissue factor levels were raised in plasma, demonstrating thereby that this via may be crucial to activate blood coagulation in patients, especially in those more severely envenomed. We also noticed that snake venom metalloproteinases accounted for most fibrinogen consumption. Our results clarify the mechanisms that activate blood coagulation during envenomation, evidencing that true intravascular coagulation syndrome, due to increased tissue factor expression, might occur during snake envenomation in human beings.

## Introduction

Snakebites, which have been considered a neglected tropical disease by the World Health Organizaton since 2009, frequently evoke hemostatic disturbances. In Brazil, *Bothrops* snakes account for approximately 20000 snakebites annually [1]. Patients usually develop local inflammatory reactions at the site of the bite, *e.g*., edema, local pain, ecchymosis, petechiae and necrosis, and also systemic bleeding manifestations, including gingival bleeding, hematuria, purpura, epistaxis, hemoptysis, among others. Thrombocytopenia, platelet dysfunction, and coagulation disorders are major laboratory findings observed in victims of *Bothrops jararaca* bites [2]–[4].

Eagle in 1937 [5] was the first researcher to notice that *B. jararaca* venom (BjV) contained at least two different principles that promoted the direct conversion of fibrinogen into fibrin, as well as the activation of prothrombin into thrombin, without the need of calcium or platelets. Snake venom metalloproteinases (SVMP) and serine proteinases (SVSP), the two main protein families found in BjV with anti-hemostatic activity [6], have been implicated in the hemostatic disorders associated with envenomation [7]. SVMP present in *Bothrops* venoms belong to a zinc-dependent enzyme family, which contributes to the inflammatory, proteolytic, hemorrhagic and procoagulant (prothrombin and factor X activators) activities in snake venoms [8]–[10]. Na_2_-EDTA completely inactivates the enzymatic activity of SVMP by chelation of divalent cations. The second most abundant enzyme class in BjV is SVSP [6], which have a highly reactive serine residue. SVSP have been reported to affect platelet aggregation, blood coagulation and fibrinolysis, and several SVSP purified from BjV show anti-hemostatic activities [11]. Serine-modifying reagents, such as 4-(2-aminoethyl)benzenesulfonyl fluoride hydrochloride (AEBSF), are irreversible serine proteinase inhibitors [12].

The current model that explains how coagulant snake venoms promote consumptive coagulopathy was published more than one hundred years ago [13]. After the initial report by Felice Fontana in 1781 [14] that venom injection into animals caused paradoxical effects – *i.e.*, an initial phase of intravascular coagulation followed by a phase of blood incoagulability –, the mechanisms whereby this phenomenon occurred were not explained until the publication of the original observations of Mellanby in 1909 [13]. He noticed that rapid injection of small quantities of *Notechis scutatus* or *Echis carinata* (*sic*) venom caused massive intravascular coagulation, in virtue of the rapid production of thrombin and enormous production of fibrin; on the other hand, slow injection generated low quantities of thrombin that in turn produced gradual fibrinogen consumption. The complete consumption of plasma fibrinogen caused incoagulability, which was restored as fibrinogen levels augmented.

Although various proteins isolated from BjV have been reported to cause systemic and local manifestation when injected into animals, no genuine approach has been used to evaluate the repercussion of local mediators generated or released at the site of venom inoculation on the induction of hemostatic disorders observed *in vivo*. Tissue factor (TF), a 47-kDa transmembrane protein, is the cellular receptor for plasma factor VII/VIIa, and thus is an essential component for initiating blood coagulation *in vivo*. In steady state conditions, TF is usually excluded from the vascular compartment, and constitutive TF expression occurs particularly in vascular smooth muscle cells, adventitial fibroblasts and pericytes. In endothelial cells, monocytes and platelets, *i.e*., cells in continuous contact with the bloodstream, TF is minimally expressed or is in an encrypted form; however, stimulation of these cells by various inflammatory mediators induces TF protein expression and activity *in vitro*. Although controversial, TF decryption has been attributed to the oxidoreductase protein disulfide isomerase (PDI) [15], [16]. Interestingly, PDI has also been reported to be present in snake venom glands [17], [18], and may therefore be present in snake venoms [19]. Thus, snake venoms, by damaging tissues locally or systemically, and by promoting the activation of circulating platelets, endothelial cells and monocytes, might induce the expression and release of TF in bloodstream, resulting in the activation of blood coagulation. However, such a mechanism of coagulation activation has never been addressed in snake envenomation.

Since fibrinogen consumption, thrombocytopenia, and secondary fibrinolysis are major hemostatic disturbances frequently observed in snakebite victims, the main objective of this study was to investigate the mechanisms that lead to the genesis of these laboratory signs in bites inflicted by *B. jararaca.* Furthermore, we evaluated whether TF levels were augmented in plasma and tissue samples obtained from animals during envenomation. We demonstrate that SVMP play a pivotal role in venom-induced coagulopathy and that the importance of TF release in plasma has been hitherto underestimated.

## Materials and Methods

### Materials

Lyophilized venom from adult specimens of *B. jararaca* snakes was obtained from the Laboratory of Herpetology, Butantan Institute. BjV was dissolved in sterile saline immediately before use. AEBSF, 1,10-phenanthroline (o-phe), bovine serum albumin (BSA), N-benzoyl-D,L-arginine-*p*-nitroanilide hydrochloride (BAPNA), and bovine thrombin were purchased from Sigma (USA), and sodium ethylenediamine tetraacetic acid (Na_2_-EDTA) from Bio-Rad (USA). Aprotinin (Trasylol) was obtained from Bayer (Brazil). To obtain rabbit anti-rat fibrinogen IgG, one rabbit was immunized i.m. with 500 µL of rat fibrinogen (4.32 mg/mL, [20]) emulsified in 500 µL of Marcol-Montanide adjuvant; at fortnight intervals, the rabbit received four additional boosters in the same adjuvant. Anti-rat fibrinogen IgG was purified and biotinylated as previously described [21]. Rabbit anti-BjV serum was obtained as described elsewhere [22]. Rat thromboplastin was prepared as described elsewhere [23]; briefly, dried thromboplastin was diluted in saline (40 mg/mL), maintained at 50°C for 20 min, and then refrigerated at 4°C overnight; the supernatant was used in clotting assays. All other reagents were of analytical grade or better.

### Ethics statement

Male Wistar rats, weighing 220–250 g, and two male 3.0-kg New Zealand rabbits, were obtained from the Animal House of Butantan Institute; they were supplied with free access to food and water. All procedures involving the use of animals were approved by the Animal Ethical Committee of Institute Butantan (protocols 142/03 and 685/09) and were in accordance with the Guide for Care and Use of Laboratory Animals (2011) and the International Guiding Principles for Biomedical Research Involving Animals (2012). Rats were anesthetized by intraperitoneal administration of xylazine (10 mg/kg b.w.)/ketamine chlorohydrate (100 mg/kg b.w.). Prior to exsanguination, immunized rabbits were anesthetized with sodium thiopental (50 mg/kg, i.v.), and blood was collected through puncture of the carotid artery.

### Inhibition of SVMP and SVSP

SVMP and SVSP were inhibited by incubation with 13 mM Na_2_-EDTA and 4 mM AEBSF, respectively. In brief, 269 mM Na_2_-EDTA (52 µL) or 200 mM AEBSF (19 µL) was added to BjV solution (1 mL, 1 mg/mL), and incubated for 1 h at 37°C. As a control of inhibition, aliquots of saline were incubated with BjV, under the same conditions. In preliminary experiments, the effectiveness of o-phe in inhibiting SVMP was also tested. An aliquot (26 µL) of 500 mM o-phe in ethanol was incubated with BjV solution, identically as described previously, and for control experiments, the same volume of vehicle was used. The effectiveness of Na_2_-EDTA in blocking the catalytic activity of SVMP was checked by assaying the minimum coagulant dose (MCD) [20] on citrated rabbit plasma, which is completely dependent on the coagulant activity of BjV metalloproteinases [24]. Clotting times were measured on a Start4 coagulometer (Stago, France). Estimates and the associated uncertainty, expressed as a 95% confidence interval [25], of MCD were calculated by linear regression analysis in Stata (version 8.0, USA), using logarithmic transformation of data. To test whether AEBSF, Na_2_-EDTA or o-phe blocked the catalytic activity of SVSP in BjV, the chromogenic substrate BAPNA was used [20]. Since BAPNA is hydrolyzed by snake venom serine proteases after the arginyl residue, we used it to detect the residual activity of SVSP in BjV.

### Envenomation protocol and sample collection

Animals were injected with aliquots of freshly-treated BjV, as described above, at the doses of 1.6 mg/kg b.w (s.c.) or 100 µg/animal (i.v.). BjV doses were selected based on previous tests, and they reproduced the acute hemostatic disturbances characteristic of *B. jararaca* envenomation. Rats injected with saline-treated BjV or saline alone (vehicle) were used as positive or negative controls, respectively. To study acute hemostatic disturbances evoked by BjV, rats were anesthetized after 3 and 6 h, and blood was collected by puncture of the abdominal aorta and dispensed in plastic bottles containing anticoagulants.

For complete blood counts, blood (500 µL) was collected into plastic bottles containing 5 µL of 269 mM Na_2_-EDTA and 5 µL of *Bothrops* antivenin (Institute Butantan, lot 1005107/C). Blood counts were determined in an automated cell counter BC-2800 Vet (Mindray, China). To obtain plasma samples, blood (4.3 mL) was collected into plastic bottles containing 700 µL of CTAD anticoagulant (75 mM trisodium citrate, 42 mM citric acid, 139 mM dextrose, 15 mM theophylline, 3.7 mM adenosine, 0.2 mM dipyridamole, and 2 µM imipramine) [7] and 50 µL of *Bothrops* antivenin, and centrifuged at 2500 *g* for 15 min at 4°C. Serum samples were obtained by maintaining blood (500 µL) without anticoagulant or antivenin at 37°C for 2 h, followed by centrifugation as mentioned above.

One circular 4-cm diameter skin fragment, whose center was the point of BjV inoculation (s.c. route), and one lung fragment (s.c. and i.v. routes) were also removed from each animal. Skin samples were sliced and used to determine BjV-induced hemorrhage, TF activity and protein expression, and PDI protein expression. TF activity and protein expression were also evaluated in lung samples. Skin (6.3 cm^2^) and lung samples (100 mg) were immediately immersed in RIPA buffer (50 mM Tris-HCl, 150 mM NaCl, 1% Triton X-100, 1% sodium deoxycholate, 0.1% SDS, pH 7.5, containing 2 mM Na_2_-EDTA, 2 mM AEBSF, 2 µM aprotinin, 130 µM bestatin hydrochloride, 28 µM E-64 and 22 µM leupeptin), and frozen at −80°C. Tissues were macerated in an IKA T10 disperser (Staufen, Germany), and frozen in dry ice and thawed in a water bath at 37°C for three times. The resultant emulsion was centrifuged at 13000 *g* for 10 min, and supernatants were frozen at -80°C until use.

### Assays

Plasma fibrinogen [26], and hemorrhage in skin samples [27] were assayed as described elsewhere. TF activity in plasma, lung and skin samples was evaluated with Actichrome TF kit (American Diagnostica, USA), according to manufacturer's instructions. In the case of tissue samples, the protein content was standardized to 1.95 mg/mL by the bicinchoninic acid protein method [28] prior to assays.

Fibrin(ogen) degradation product (FDP/fdp) levels in plasma were evaluated by a home-made double-antibody sandwich ELISA assay, based on a previous protocol [21], using rabbit anti-rat fibrinogen IgG for coating, rat fibrinogen (3.9-1000 ng/mL) as standard, and biotinylated rabbit anti-rat fibrinogen IgG. To remove residual fibrinogen from plasma, samples (200 µL) were initially incubated with aprotinin (10000 U/mL, 10 µL) and thrombin (30 U/mL, 200 µL) for 15 min at 37°C, and centrifuged at 10000 *g* for 15 min.

Prothrombin time was assayed by incubating plasma samples (80 µL) with rat thromboplastin (40 µL) for 1 min at 37°C, and then 50 mM CaCl_2_ (40 µL) was added and clotting times were measured. Factor VII (FVII) coagulant activity was determined using FVII-deficient plasma (HemosIL, USA, 40 µL) incubated with plasma samples (40 µL, 1/50 dilution) and rat thromboplastin (40 µL) at 37°C for 1 min; clotting time was measured after the addition of 50 mM CaCl_2_ (40 µL). A standard curve was constructed by using a pool of normal rat plasma diluted from 1/10 to 1/800, and considering the 1/50 dilution as 100% of FVII. All clotting times were measured on a Start4 coagulometer.

Circulating BjV levels in serum was assayed by a modification of a procedure previously described [29]. Briefly, Nunc 96-well microplates were coated with commercial *Bothrops* antivenin (100 µg/mL, Institute Butantan, lot 1001103/D), and blocked with 3% BSA in carbonate buffer, pH 9.6. Then, 100 µL of diluted serum samples (1/10 in incubation buffer [PBS containing 1% BSA and 0.05% Tween 20]) or venom standards (1.95-500 ng/mL BjV diluted in incubation buffer containing 10% of a pool of normal rat serum) were added to wells. Subsequently, rabbit anti-BjV serum (1/1000), and goat anti-rabbit IgG-peroxidase antibody were used, and reaction was developed using *o*-phenylenediamine.

### TF and PDI protein expression

TF and PDI protein expression in skin samples (50 µg protein/lane) was evaluated by western blotting. Briefly, proteins were electrophoresed under reducing conditions in 12% SDS-PAGE gels [30] and transferred onto 0.2-µm nitrocellulose membranes. Subsequently, membranes were blocked, incubated at room temperature for 2 h with either a 1∶1000 mouse monoclonal anti-TF antibody (TF9-10H10, Calbiochem, USA) or 1/10000 rabbit polyclonal anti-PDI antibody (Sigma P7372) in blocking solution, washed, and subsequently incubated with 1/10000 peroxidase-conjugated anti-mouse IgG (Sigma A4416) or anti-rabbit IgG (Sigma A0545). Expression of glyceraldehyde 3-phosphate dehydrogenase (GAPDH), used as a loading control, was evaluated using a peroxidase-conjugated anti-GAPDH antibody (Sigma G9295). Membranes were developed as reported elsewhere [20], scanned with resolution of 300 dpi, and densitometric analyses were done with TotalLab TL100 software (USA). For relative quantification [31], optical densities of bands (volumes) were divided respectively by the total optical density of lanes in membranes stained with Ponceau S. One sample from a saline-injected animal was used as an internal control throughout experiments, and it was considered as 1 for determining relative expression of protein bands.

### Statistical analyses

The efficiency of preincubation of BjV with inhibitors, routes of BjV administration, and time periods were compared using ANOVA, followed by the Tukey test. TF activity in lung and skin samples was compared by Student's t test. Whenever necessary, data transformation was undertaken to obtain homocedasticity and normal distribution. Statistical analyses were performed using the softwares SigmaStat (version 3.5, USA) and Stata (version 8.0, USA). Differences with p<0.05 were considered statistically significant. Data were expressed as mean ± standard error of mean (s.e.m.).

### Accession numbers

Accession numbers for proteins studied herein, according to UniProtKB/Swiss-Prot database, are: tissue factor (P42533); protein disulfide isomerase (P04785), fibrinogen (P06399, P14480, P02680), hemoglobin (P01946, P02091), and factor VII (Q8K3U6).

## Results

### SVMP and SVSP are blocked by incubation with Na_2_-EDTA and AEBSF, respectively

In this study, we used the specific inhibitor AEBSF to inhibit serine proteinases. In order to block the enzymatic activity of SVMP in the venom, we initially compared two non-specific inhibitors largely used in toxinology research, Na_2_-EDTA and 1,10-phenanthroline (o-phe). Incubation of BjV with AEBSF inhibited the amidolytic activity of SVSP by 93%, whereas neither Na_2_-EDTA nor o-phe importantly blocked SVSP (Table 1). We also examined the efficiency of inhibitors in blocking the coagulant activity of BjV in rabbit plasma, which is almost exclusively dependent on procoagulant activators of BjV [24]. In rabbit plasma, the MCD of BjV incubated with saline was 3.6±1.4 µg/mL, and Na_2_-EDTA reduced this activity by *ca*. 70-fold (MCD = 262.4±1.8 µg/mL). Incubation with o-phe also diminished the clotting activity of BjV by approximately 40-fold (MCD  = 336.7±2.0 µg/mL vs. MCD  = 7.9±1.3 µg/mL for BjV incubated with ethanol, the vehicle for o-phe). In contrast, AEBSF did not inhibit the coagulant activity of BjV (MCD = 4.0±1.4 µg/mL). Altogether, these results demonstrated that preincubation of BjV with either Na_2_-EDTA or o-phe inhibited SVMP, but not SVSP activity, whereas AEBSF markedly inhibited only SVSP activity.

**Table 1:** Hydrolysis of the chromogenic substrate BAPNA by *B. jararaca* venom, incubated or not with inhibitors of serine proteinase and metalloproteinases.

Treatment	Inhibition (%)
BjV + buffer*	0.0±0.0
BjV + 4 mM AEBSF	93.3±0.1
BjV+ 13 mM Na_2_EDTA	0.0±0.0
BjV + 13 mM o-phe	3.3±0.1
BjV+ ethanol (control)^†^	5.1±0.4

*Specific activity  =  48.3±0.3 nmol p-nitroaniline/min/mg venom (triplicate determinations of two different experiments). ^†^Since o-phe was diluted in ethanol, BjV was incubated with the same volume of vehicle to determine the extent to which BAPNA hydrolysis was inhibited by the solvent. Results are expressed as mean ± s.e.m.

In preliminary experiments, we also evaluated whether o-phe and Na_2_-EDTA produced similar *in vivo* results, in order to choose one SVMP inhibitor for subsequent experiments. The results obtained for platelet count and fibrinogen assay at 3 h showed that both Na_2_-EDTA and o-phe provided similar results and experimental profiles (Figure S1). Based on these results, Na_2_-EDTA was preferred since it dissolved in aqueous solution, and no additional group was required for vehicle controls.

### Venom serum levels are similar in envenomed groups

In snakebites, venom is usually injected into victims via s.c. or i.m. routes. In order to assess whether local hemorrhage and an inflammatory reaction could modify the systemic hemostatic manifestations evoked by BjV, the i.v. and s.c. routes were used to compare hemostatic parameters in the acute phase of envenomation (3 and 6 h). Intravenous injection of 100 µg/animal defibrinogenated 100% of rats, and caused no clinical manifestations. After previous experiments, we elected the s.c. dose of 1.6 mg/kg b.w., since hemostatic disturbances at 3 and 6 h were similar to those observed in patients on admission to hospital, and animals behaved normally. Using this dose, fibrinogen levels and platelet counts were progressively restored after 8 h, so that at 24 h they were hemostatically recovered (mean platelet counts are higher than 600×10^9^/L and mean fibrinogen levels are higher than 100 mg/dL, data not shown).

**Figure 1:**
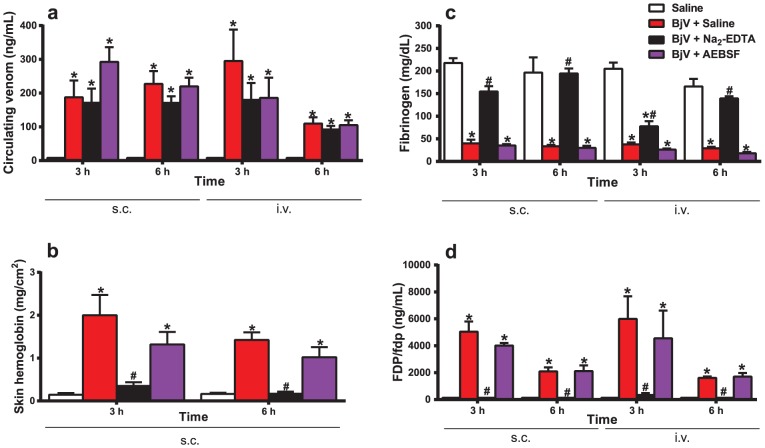
Circulating venom levels (a), local hemorrhage (b), plasma fibrinogen levels (c), and serum FDP/fdp levels (d) in rats 3 and 6 h after BjV administration. Rats were injected s.c. (1.6 mg/kg) or i.v. (100 µg/animal) with venom incubated with saline (BjV+Saline), 13 mM Na_2_-EDTA (BjV+Na_2_-EDTA) or 4 mM AEBSF (BjV+AEBSF). Control animals were treated under the same conditions, but were injected with saline (Saline). *p<0.005 compared with saline-treated rats (Saline). ^#^p<0.005 compared with BjV+Saline. Data are expressed as mean ± s.e.m (n = 5–6/group).

Initially, circulating BjV levels were measured to test whether preincubation of BjV with Na_2_-EDTA or AEBSF modified the absorption of BjV from tissues into bloodstream, and could thereby interfere with subsequent analyses. Rats injected with BjV, regardless of the treatment or route used, exhibited statistically significant increases in venom levels compared with the saline group at 3 and 6 h (Fig. 1a). Although some fluctuation was noticed in venom levels, no statistically significant difference was observed between the results of the Na_2_-EDTA- or AEBSF-treated groups compared with saline-treated BjV group (p = 0.897), both for 3 and 6 h. These results confirmed that neither Na_2_-EDTA nor AEBSF prevented BjV from entering the bloodstream, nor altered the levels of circulating BjV. When animals received BjV i.v., circulating levels cleared more rapidly at 6 h (p = 0.017).

### Na_2_-EDTA inhibits local hemorrhage, hypofibrinogenemia and increased FDP/fdp levels

As expected, subcutaneous injection of BjV into rats resulted in local hemorrhage (Fig. 1b). Preincubation of BjV with AEBSF decreased the extent of local hemorrhage by approximately 30%. Na_2_-EDTA markedly reduced (around 85%) local hemorrhage at 3 and 6 h compared with saline-treated BjV (p<0.001). Furthermore, BjV induced a remarkable drop in plasma fibrinogen levels and a simultaneous sudden increase in FDP/fdp levels at 3 and 6 h (p<0.001), independently of the route of administration (Fig. 1c,d). Preincubation with Na_2_-EDTA inhibited fibrinogen consumption at 3 h (p<0.001 for s.c, and p<0.004 for i.v. route), and more markedly at 6 h (p<0.001 for both routes). In addition, Na_2_-EDTA-treated BjV was less effective in reducing fibrinogen consumption at 3 h when given i.v. compared to s.c. However, AEBSF-treated BjV evoked the same extent of fibrinogen consumption as that of saline-treated BjV both at 3 and 6 h (Fig. 1c). Likewise, the rise in FDP/fdp levels was promptly reversed by preincubation of BjV with Na_2_-EDTA, but not by AEBSF (Fig. 1d). Together, these results show that the preincubation of BjV with Na_2_-EDTA drastically inhibited local hemorrhage, fibrinogen consumption and fibrinolysis activation, suggesting that SVMP have an essential role in inducing coagulopathy during envenomation.

### Thrombocytopenia is inhibited by neither Na_2_-EDTA nor AEBSF

**Figure 2:**
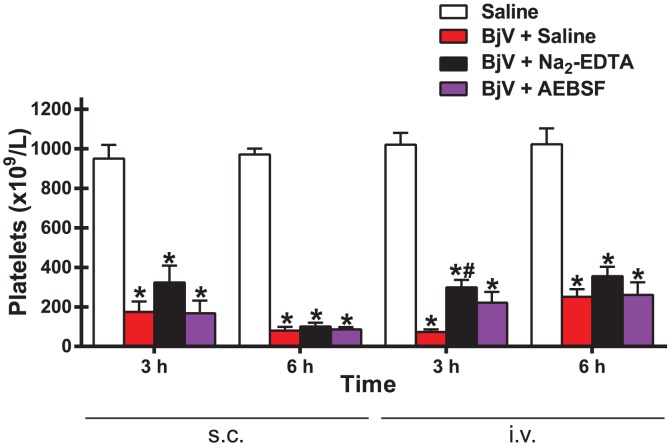
Platelet counts in rats 3 and 6 h after BjV administration. Rats were injected s.c. (1.6 mg/kg) or i.v. (100 µg/animal) with venom incubated with saline (**BjV+Saline**), 13 mM Na_2_-EDTA (**BjV+Na_2_-EDTA**) or 4 mM AEBSF (**BjV+AEBSF**). Control animals were treated under the same conditions, but were injected with saline (**Saline**). *p<0.002 compared with saline-treated rats (**Saline**). ^#^p<0.001 compared with **BjV+Saline**. Data are expressed as mean ± s.e.m (n = 5–6/group).

Independently of the route of venom administration, platelet counts decreased markedly (around 80–90%) in rats receiving BjV at 3 and 6 h (p<0.001) (Fig. 2). In addition, regardless of the preincubation used, platelet counts at 6 h were lower when BjV was given s.c. than when given i.v., perhaps because of the lower dose of venom injected and therefore a faster clearance of circulating venom. Neither AEBSF nor Na_2_-EDTA conspicuously attenuated the drop in platelet count, although platelet counts tended to be somewhat higher with Na_2_-EDTA treatment for the i.v. group at 3 h. These data demonstrate that neither SVMP nor SVSP were involved in venom-induced thrombocytopenia.

### Administration of BjV increases TF activity in the circulation, and TF protein expression in skin and lungs

**Figure 3:**
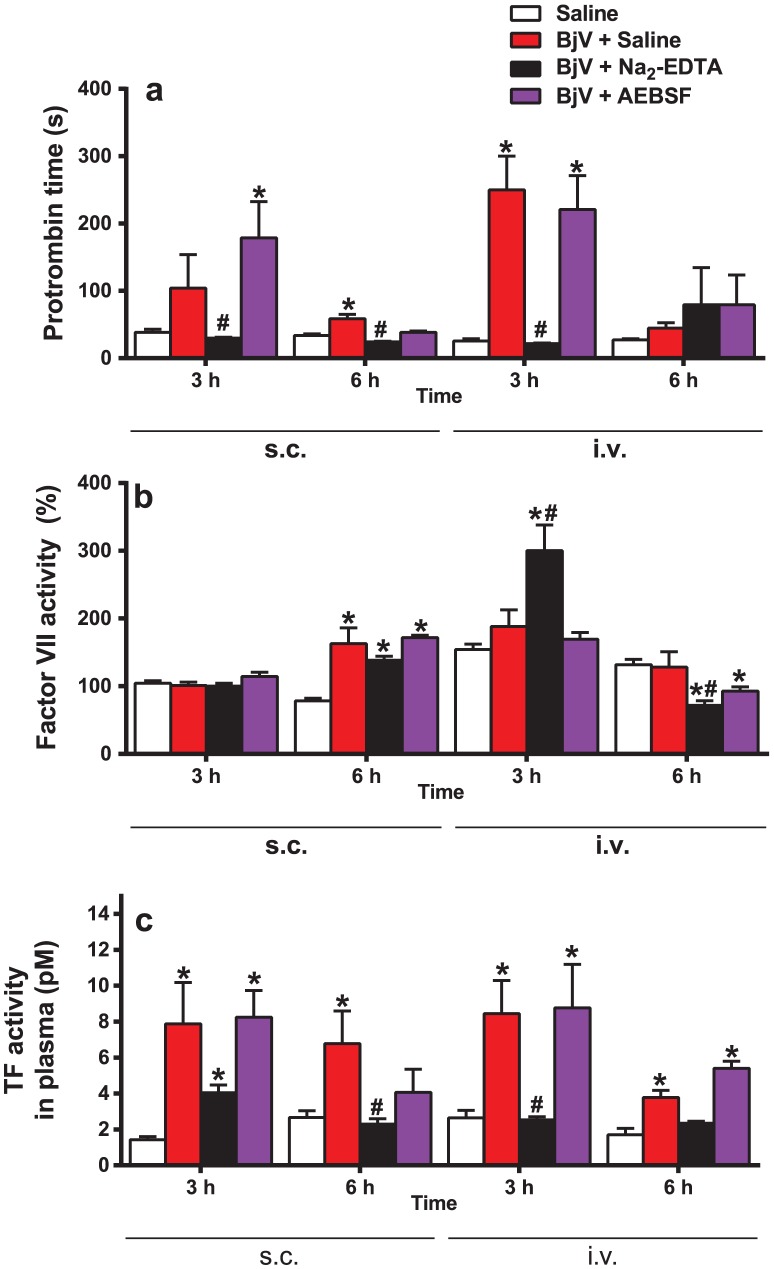
Prothrombin time (a), factor VII coagulant activity (b) and plasma TF activity (c) in rats 3 and 6 h after BjV administration. Rats were injected s.c. (1.6 mg/kg) or i.v. (100 µg/animal) with venom incubated with saline (BjV+Saline), 13 mM Na_2_-EDTA (BjV+Na_2_-EDTA) or 4 mM AEBSF (BjV+AEBSF). Control animals were treated under the same conditions, but were injected with saline (Saline). *p<0.04 compared with saline-treated rats (Saline). ^#^p<0.05 compared with BjV+Saline. Data are expressed as mean ± s.e.m (n = 5–6/group).

Initially, PT was used to check activation of the extrinsic pathway of coagulation. As expected, PT was markedly prolonged in rats injected with saline-treated BjV at 3 h, and this increase in PT tended to subside at 6 h, for both routes of venom administration. Na_2_-EDTA abolished this increase (Fig. 3a), except at 6 h for the i.v. route, because PT had already returned to basal levels in all groups; in contrast, AEBSF had no important effect on PT. FVII levels (Fig. 3b) were not reduced during envenomation, except at 6 h in rats treated with Na_2_-EDTA or AEBSF by the i.v. route; minor increases were noticed at 6 h in rats injected s.c. with BjV, regardless of the preincubation, and there was a major increase at 3 h in those receiving Na_2_-EDTA-treated BjV i.v. These data show that FVII consumption is apparently not an important event during envenomation, and that hypofibrinogenemia may be the primary cause of prolongation of PT. However, since FVII plasma levels are elevated during stressful conditions [32], consumption might be masked by a simultaneous increase in the synthesis of this factor. Thus, to investigate whether the inoculation of BjV into rats induced a rise in plasma TF levels, a TF activity assay was employed. As shown in Figure 3c, rats injected with BjV showed a marked increase in plasma TF levels, in comparison with saline-injected animals (p = 0.01). Interestingly, Na_2_-EDTA, but not AEBSF, mitigated this increase (p<0.05). The i.v. and s.c. administration of BjV showed increased levels of TF activity in plasma, demonstrating that the local reaction, induced by s.c. injection, did not completely account for the rise in TF activity in plasma.

**Figure 4:**
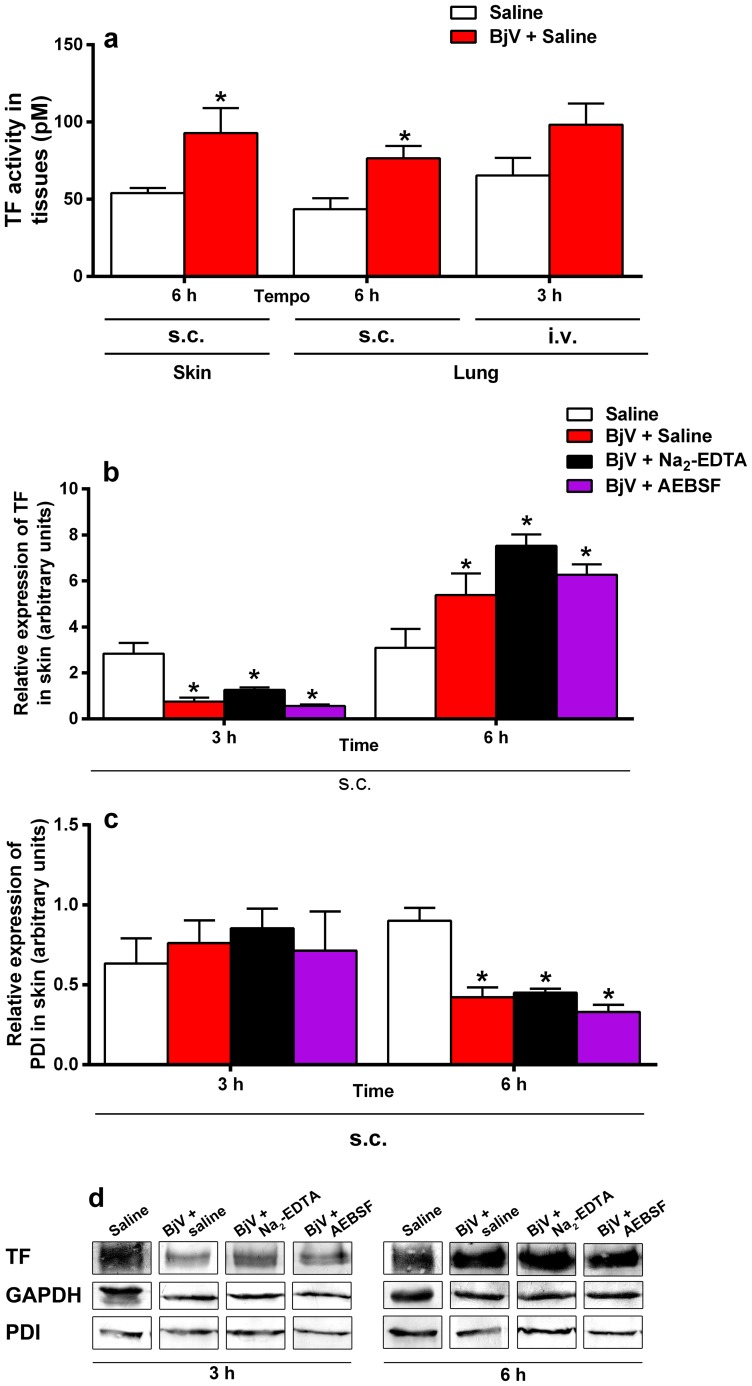
TF activity in skin and lung (a), and protein expression of TF (b) and PDI (c) in skin from rats 3 and 6 h after BjV administration. Rats were injected s.c. (1.6 mg/kg) or i.v. (100 µg/animal) with venom incubated with saline (BjV+Saline), 13 mM Na_2_-EDTA (BjV+ Na_2_-EDTA) or 4 mM AEBSF (BjV+AEBSF). Control animals were treated under the same conditions, but were injected with saline (Saline). *p<0.04 compared with saline-treated rats (Saline). Data are expressed as mean ± s.e.m (n = 5–6/group). (d) Typical results obtained from western blottting analysis of TF (47 kDa), PDI (57 kDa), and GAPDH (37 kDa, internal control) bands from skin homogenates at 3 and 6 h after venom or saline injection. The intensity of the bands was quantified by densitometry and the values obtained from the analysis of 5–6 individuals are shown in figures b and c.

To investigate where TF was being expressed, we analyzed samples from skin and lungs. After s.c. injection, high levels of TF activity were noticed in skin (p = 0.048) and lung (p = 0.015) at 6 h (Fig. 4a). On the other hand, i.v. showed a trend for high levels in lung, but no statistically significant difference was observed. Thus, to understand whether elevated TF activity resulted from an increased protein expression in lung and skin tissues, semiquantitative western blotting was used to evaluate TF and PDI protein expression. Protein bands of 47 and 57 kDa, corresponding to TF and PDI, respectively, were observed in skin samples. In lung tissue, no bands were noticed, probably because the amount of PDI and TF proteins in the samples was below the detection limit of western blotting (data not shown), and therefore only skin samples were analyzed further. Figure 4b,d depicts a statistically significant fall in TF protein expression in skin at 3 h, independently of the treatment used for BjV, in comparison with normal tissue (p<0.001). Inversely, TF expression was augmented (p = 0.014) at 6 h in animals that received BjV in comparison with those that received saline; however, no statistically significant difference was noticed in TF expression among groups that received BjV pretreatments. On the other hand, PDI expression (Fig. 4c,d) was constant at 3 h, but a remarkable drop was noticed in all groups that received BjV at 6 h (p<0.001). GAPDH protein expression (Fig. 4d) was also used as a control, and no statistically significant difference was noted among groups at 3 or 6 h (data not shown).

## Discussion

BjV is a rich source of proteins and enzymes that destabilize hemostasis. We reasoned that exposure, expression and/or release of TF induced by BjV might occur at the site of venom inoculation or systemically, in virtue of distant tissue damage evoked by venom toxins. To date, augmented TF expression has never been demonstrated in snakebites, although it has been claimed, without scientific evidence, that it does not occur [33]. We verified for the first time that a marked increase in TF levels occurs in plasma and tissues of envenomed rats. This evidence suggests that coagulopathy is not only due to the direct activity of snake venom toxins on coagulation factors, as demonstrated elsewhere [13], but also by augmented TF expression and release. Irrespective of the location where TF is released, our results demonstrate that TF expression/activity is increased during envenomation, and may trigger the blood coagulation cascade following snakebite. However, the intensity and relevance of this finding to the overall picture of hemostatic dysfunction requires further investigation.

In lieu of employing purified proteins [34], we elected to use BjV, which contains a wide variety of toxins that act interactively, to evaluate whether SVMP and SVSP were important to induce hemostatic disturbances. Surprisingly, SVSP had no major role in *B. jararaca*-induced hemostatic disturbances. Only preincubation of venom with Na_2_-EDTA was able to substantially inhibit fibrinogen consumption, PT prolongation, FDP/fdp generation, local hemorrhage, and the increase in TF levels. Results obtained for the incubation of BjV simultaneously with Na_2_-EDTA and AEBSF were not different from those reported for Na_2_-EDTA alone (data not shown). Local hemorrhage, which is usually attributed to the activity of SVMP [35], is frequently observed in envenomed patients, and, as expected, was abrogated by treatment of BjV with Na_2_-EDTA, as reported previously in mice [36]. On the other hand, Na_2_-EDTA minimally blocked BjV-induced thrombocytopenia. Using batismastat, clodronate, and doxycycline to inhibit SVMP from *Bothrops asper* venom, similar conclusions were reached about the pivotal role of SVMP in venom-induced coagulopathy and hemorrhage, and their lack of involvement in thrombocytopenia in mice [34], [37], [38].

Fibrinogen consumption has been hypothesized to be the consequence of the direct defibrinogenating activity of thrombin-like enzymes, and/or of generation of intravascular thrombin promoted by prothrombin and factor X activators found in BjV [24]. Our findings show that SVMP play an essential role in inducing fibrinogen consumption in rats, and that the direct action of thrombin-like enzymes (SVSP) on fibrinogen contributes minimally to defibrinogenation after s.c. injection. In Brazil, most physicians and toxinologists immediately associate blood incoagulability observed in either humans or animals bitten by *Bothrops* snakes with the action of thrombin-like enzymes. This assumption is based on early reports, particularly from those that described the coagulant activity of *Bothrops* venoms [39]–[42] or that isolated thrombin-like enzymes [43]–[47]. Among the broad variety of coagulant enzymes found in snake venoms, various investigations have focused their research on isolating and characterizing thrombin-like enzymes, given the simplicity of isolating fibrinogen, the most abundant coagulation factor found in plasma. However, Eagle [5] had already observed that BjV, not only contained enzymes that clotted fibrinogen, whose action was similar to thrombin, but also prothrombin-activating enzymes. On the other hand, Gastão Rosenfeld, an eminent physician who worked at Hospital Vital Brazil, in Institute Butantan, characterized the coagulant and hemolytic activity of animal venoms in Brazil [41]. His teachings and paradigms still reverberate in Brazil, and physicians and scientists continue to believe that thrombin-like enzymes are the main enzymes involved in defibrinogenation in bites inflicted by *Bothrops* snakes. According to Rosenfeld et al. [48, page 244], “Among bothropic venom, *B. jararaca* and *B. atrox* were more extensively studied than others. No disagreement exits with respect to their thrombinlike activity (Janszky, 1950, 1956; G. Rosenfeld et al., 1959; Nahas et al., 1964), which is responsible for the defibrination syndrome in the clinic. As a consequence of fibrinogen depletion, blood remains incoagulable”. This conclusion has prevailed henceforth, and no investigation has rigorously examined it. However, other signs apparentely strengthened this paradigm. For example, the cause of blood incoagulability in patients bitten by South American rattlesnakes (*Crotalus durissus* spp), whose venom contains exclusively thrombin-like enzymes [49], resembled the defibrinogenation evoked by *Bothrops* snakes. As a result, thrombin-like enzymes were erroneously considered as the main toxins that elicit fibrinogen consumption in the latter envenomation. Consolidation of this paradigm has been reinforced by the use of thrombin-like enzymes for the treatment of thromboembolic diseases [50], [51], which promote safe defibrinogenation, similar to that observed in most snakebites.

However, our results do not agree with the traditional assumption that thrombin-like enzymes are the main toxins accounting for defibrinogenation, and indicate that SVMP are crucial enzymes promoting fibrinogen consumption, at least in rabbits [7], [24], mice [38], and rats. However, which class of toxins accounts for most fibrinogen consumption in humans remains to be investigated, but we demonstrate herein that procoagulant enzymes and high TF plasma levels may exert a relevant role in the hemostatic disorder evoked by *Bothrops* bites, since intravascular thrombin generation, evidenced by raised plasma levels of TAT complex [52], [53], are noticed in patients.

In *Bothrops* sp. venoms, few isolated SVMP and SVSP have been reported to interfere with platelet function and/or cause thrombocytopenia [34], [54]–[60]. SVMP inhibition did not protect rats from the drop in platelet count observed during *B. jararaca* envenomation. Likewise, treatment of *B. asper* or *Bothrops caribbaeus* venom with SVMP inhibitors could not block thrombocytopenia [34], [61]. Thus, this evidence suggests that SVMP have a minor role in directly inducing thrombocytopenia in rats, and that other pathophysiological mechanisms are involved in thrombocytopenia in *B. jararaca* envenomation. Interestingly, our findings indicate that SVSP reported to activate platelets *ex vivo* [57] did not seem to be important *in vivo*. The C-type lectin aspercetin, similar to botrocetin found in *B. jararaca* venom [62], is apparently crucial to induce thrombocytopenia in mice injected i.v. with *B. asper* venom [34]. Whether botrocetin or other C-type lectins found in BjV account for thrombocytopenia *in vivo* is a matter for future investigation.

Laboratory data from victims of *B. jararaca* snakebites show no correlation between fibrinogen consumption and thrombocytopenia [52]. Our findings corroborate such clinical observations, and do indicate that diverse and complex pathophysiological mechanisms are involved in this process. In line with this assumption, variation in the composition of toxins during the ontogenetic development of *B. jararaca* snakes [20], [63] may explain why patients bitten by young snakes have a higher incidence of blood incoagulability and a trend to higher platelet counts on admission in hospital, compared to those bitten by adult snakes, who have a more accentuated fall in platelet counts and a lower frequency of blood incoagulability [2], [64]. These findings demonstrate that there is no single mechanism or main toxin that may explain all events occurring in envenomation by *B. jararaca.*

Local injury was reported to play a prominent role in sequestering platelets after s.c. or i.m. venom inoculation [65]. Two routes were used here to inoculate BjV, so that we could study the contribution of the local lesion to systemic hemostatic disturbances. BjV induced intense proteolytic activity and inflammatory reaction at the site of venom inoculation, demonstrated by the presence of intense local hemorrhage. However, animals injected with Na_2_-EDTA-treated BjV showed minimal local injury, but still demonstrated high platelet consumption, indicating that the local lesion minimally contributes to the sequestration of platelets from the circulation.

Interestingly, neither Na_2_-EDTA nor AEBSF interfered with the kinetics or levels of BjV in circulation, although we have evaluated venenemia in only two time intervals. Anai et al. [66] reported that preincubation of *B. jararaca* venom with polyclonal antibodies anti-jararafibrase I (*i.e*., jararhagin [67]) neutralized the hemorrhagic activity of crude *B. jararaca* venom, and prevented the development of hemostatic disturbances *in vivo*, demonstrating that hemorrhagic SVMP facilitate diffusion and absorption of coagulant toxins into the circulation. However, the mechanisms of inhibition of SVMP by antibodies and Na_2_-EDTA are different, since the latter directly inhibits the catalytic activity of SVMP, whereas the former bind to diverse epitopes in SVMP in order to inhibit their biological activity. Thus, our results suggest that other toxins. such as hyaluronidases [68], may facilitate venom diffusion/absorption.

TF has been reported to be involved in inflammation and thrombosis [69], and several mediators, including proinflammatory cytokines and thrombin, induce TF expression [15]. In view of the intense inflammatory activity evoked by SVMP at the site of venom inoculation, tissue injury, cell necrosis/apoptosis, and/or the release of proinflammatory cytokines [70] may have accounted for the raised expression of TF in skin and lungs. Interestingly, berythractivase, a SVMP isolated from *Bothrops erythromelas* venom, but not jararhagin, from BjV, has been demonstrated to render endothelial cells highly thrombogenic *in vitro*, due to upregulation of TF activity and expression [71].

TF levels were raised in plasma as soon as 3 h after BjV injection, but statistically significant increases in skin and lung TF expression and activity were noticed exclusively at 6 h. These results suggest that activation of circulating cells, such as monocytes and platelets (two cell types known to express TF when activated [15]) by BjV might also be involved in the increase in plasma TF levels. Sustained raised levels of TF in plasma at 6 h were only noticed when BjV was administered s.c., suggesting that this route provides additional stimulus for TF production/decryption. It is difficult to explain the significant decrease in TF protein expression in skin at 3 h, but one plausible explanation could be that BjV hydrolyses TF, although this possibility was not tested here.

In skin, TF protein expression was markedly elevated at 6 h, and PDI was simultaneously diminished; however, the mechanism responsible for these findings remains to be demonstrated. Although the role of PDI in TF decryption is questionable, in models of thrombosis PDI accumulates at the site of vascular injury [16], [72]. On the other hand, inhibition of PDI at the endothelial cell surface enhances TF pro-coagulating activity by affecting phosphatidylserine exposure [73]. Thus, the decrease in protein expression in PDI at 6 h may intensify the hemostatic disturbances during envenomation.

Given the observed increase in TF protein expression in skin, lung and plasma, a drop in plasma FVII was expected. However, normal or increased FVII levels were observed here. In fact, FVII has the shortest mean-life (4–7 h) of blood coagulation factors in the circulation, and the lack of diminished FVII levels may be ascribed to an increased hepatic synthesis that may occur in stressful situations [32]. A steady decrease in FVII levels has been reported for one patient bitten by *Bothrops neuwiedi* [74], although most snakebites in humans do not induce a marked FVII consumption [75]–[78]. As shown elsewhere [42], the coagulant activity of BjV does not depend on FVII *in vitro*.

Disseminated intravascular coagulation (DIC) is characterized by a TF-mediated coagulation activation induced by cytokines, depletion of natural anticoagulants and PAI-1-mediated fibrinolysis inhibition [79]. In virtue of elevated FDP/fdp levels, thrombocytopenia, prolonged prothrombin time and fibrinogen consumption, which were also observed herein, snake envenomation has been associated with or may evolve to DIC [80]. Since high plasma TF levels have been described in patients with DIC [81], our results suggest that raised TF levels may have an important role in activating the blood coagulation cascade, especially in more severely envenomed patients, in which the local lesion is more extensive [2]. On the other hand, since hemostatic disturbances are rapidly recovered after antivenom therapy in mildly or moderately envenomed patients [2], the involvement of plasma TF in bleeding manifestations observed therein awaits clinical evaluation.

In conclusion, we show that SVMP are crucially involved in the coagulopathy evoked by BjV in rats. Since BjV-induced thrombocytopenia was not mitigated by any inhibitor, our findings demonstrate that SVMP and SVSP are not directly associated with this phenomenon, and that other mechanism(s) or BjV toxins are involved. Our findings also indicate that TF is an additional component that should be considered when discussing snake venom-induced hemostatic disturbances. Moreover, the evidence of increased TF levels in plasma and skin is extremely important in dealing with hemostatic disturbances in severely envenomed patients bitten by *B. jararaca* snakes, since it approximates the so-called DIC-like syndrome, which occurs in snake envenomation, to the true DIC syndrome, initiated by increased TF expression. Our results suggest that plasma TF levels are likely to be elevated in patients bitten by poisonous snakes, and that their levels may be correlated with the frequency and intensity of hemostatic disturbances. Therapeutic interventions in this pathway should be tested as an ancillary treatment to antivenom therapy for allowing a prompt interruption to the development of true DIC syndrome.

## Supporting Information

Figure S1 **Platelet counts (a), and plasma fibrinogen levels (b) in rats 3 h after BjV administration**. Rats were injected s.c. (1.6 mg/kg) with venom incubated with saline (**BjV+Saline**), 13 mM Na_2_-EDTA (**BjV+Na_2_-EDTA**), 4 mM AEBSF (**BjV+AEBSF**), 13 mM 1,10 phenathroline (**BjV+o-phe**) or ethanol (**BjV+Ethanol**, control group for **BjV+o-phe**). Control animals were treated under the same conditions, but were injected with saline (**Saline**). *p<0.001 compared with saline-treated rats (**Saline**). ^#^p<0.001 compared with **BjV+Saline.** ^⧫^A statistically significant difference was noticed between **BjV+Na_2_-EDTA** and **BjV+o-phe** for plasma fibrinogen (p = 0.005). Data are expressed as mean ± s.e.m (n = 5–6/group).
(EPS)
